# Perspectives on Dementia Service Use and Family Caregiving Among the Oneida Nation of Wisconsin

**DOI:** 10.1093/geroni/igaa057.2498

**Published:** 2020-12-16

**Authors:** Mary Wyman, Nickolas Lambrou, Debra Miller, Sunshine Wheelock, Florence Petri, Marlene Summers, Carey Gleason, Dorothy Edwards

**Affiliations:** 1 University of Wisconsin, Madison, Wisconsin, United States; 2 Oneida Comprehensive Health Division, Madison, Wisconsin, United States; 3 Oneida Elder Services, Madison, Wisconsin, United States; 4 Oneida Nation Commission on Aging, Madison, Wisconsin, United States; 5 University of Wisconsin School of Medicine and Public Health, Madison, Wisconsin, United States

## Abstract

Prevalence of dementia among American Indian/Alaska Natives (AI/AN) is higher than in white populations, and AI/AN communities experience dementia care service gaps. This study explored perspectives within AI/AN communities regarding dementia, the family caregiver role, and home and community-based service use. Using tenets of Community-Based Participatory Research, qualitative interviews and a brief survey were conducted with 22 members of the Oneida Nation of Wisconsin (mean age 71 years, 73% female). Of the sample, 63.6% identified as a past or current family caregiver for a loved one with dementia. Awareness of services varied; 82% were aware of memory cafes, 75% knew of the caregiver support group, and 43% were familiar with dementia care specialist services. Thematic analysis revealed shared values of involving the family and community in dementia care, and offer guidance to support greater engagement in services. Implications for culturally-tailored service provision within AI/AN communities are discussed.

